# Inflammation-Related Markers in Pediatric Psoriasis: Resistin as a Potential Marker of Psoriasis Severity

**DOI:** 10.3390/jcm14051689

**Published:** 2025-03-03

**Authors:** Magdalena Szczegielniak, Aleksandra Lesiak, Adam Reich, Aleksandra Opalińska, Bartosz Zakrzewski, Hubert Arasiewicz, Kamil Grabowski, Daniel Nolberczak, Joanna Narbutt

**Affiliations:** 1 Department of Dermatology, Pediatric Dermatology and Oncology, Medical University of Lodz, 90-419 Lodz, Poland; aleksandra.lesiak@umed.lodz.pl (A.L.); kamiljgrabowski@gmail.com (K.G.); d.nolberczak@gmail.com (D.N.); joanna.narbutt@umed.lodz.pl (J.N.); 2 Department of Dermatology, Medical College of Rzeszow University, 35-055 Rzeszow, Poland; adamandrzejreich@gmail.com (A.R.); aopalinska88@gmail.com (A.O.); 3 Zakrzewscy Clinic of Aesthetic Medicine and Dermatology, 40-246 Katowice, Poland; bartosz.zakrz@gmail.com; 4 Department of Dermatology and Vascular Anomalies, John Paul II Centre of Pediatrics, 41-200 Sosnowiec, Poland; hubert.arasiewicz@gmail.com

**Keywords:** pediatric psoriasis, homocysteine, adiponectin, pentraxin 3, resistin, leptin

## Abstract

**Background/Objective:** Psoriasis is a chronic inflammatory skin disease. Studies on adult population have confirmed that there is an association between psoriasis and metabolic as well as cardiovascular diseases. The aim of this study was to evaluate the inflammatory potential and the association of psoriasis with metabolic and cardiovascular risk by analyzing serum concentrations of homocysteine, adiponectin, resistin, leptin, and pentraxin 3 in pediatric patients with psoriasis. Additionally, the study explored correlations between these biomarkers and psoriasis severity. **Methods:** The study included 75 pediatric patients (47 girls and 28 boys) aged 2–17 years with clinically confirmed psoriasis. In addition, 28 healthy children (15 girls and 13 boys) without psoriasis, metabolic or inflammatory diseases made up the control group. Psoriasis severity was evaluated using the scales psoriasis area and severity index (PASI) and the body surface area (BSA). Serum concentrations of homocysteine, adiponectin, pentraxin 3, resistin, and leptin were measured in both groups. **Results:** Children with psoriasis exhibited higher serum levels of homocysteine, resistin, leptin, and pentraxin 3 and lower serum levels of adiponectin compared to the control group. A positive correlation was observed between resistin serum concentration and psoriasis severity. Elevated resistin levels were associated with higher PASI and BSA scores. **Conclusions:** Psoriasis is an inflammatory disease that is potentially linked to metabolic disorders. Resistin may serve as a biomarker for psoriasis severity; however, this relationship requires further research.

## 1. Introduction

Psoriasis is a chronic, recurrent skin condition with an immunological basis, characterized by alternating periods of exacerbations and remissions. It affects approximately 1–3% of the global population, with nearly one-third of cases manifesting during childhood. The prevalence of pediatric psoriasis is estimated to be 0.7–2.1%. In recent years, there has been an increase in moderate to severe forms of psoriasis in children. The onset of the disease in childhood may herald its severe course. In the pediatric population, the same subtypes of the disease are distinguished as in adults, but its clinical process differs in the individual age groups. Differences in the course of psoriasis in children compared to adults result from different structure and immaturity of the skin. The most common form of psoriasis in children is plaque type psoriasis. In pediatric patients, thinner psoriatic plaques are observed, sometimes without the presence of scales. In infants, psoriatic lesions often occur in the anogenital areas and appear as oozing, erythematous foci. Children with psoriasis are more likely to report itchy skin than adults. The diagnosis is based mainly on clinical evaluation of the morphology of skin lesions. Psoriasis can have a negative impact on the quality of life of patients and their families. Psoriasis is now considered a systemic inflammatory disease contributed by multiple biochemical pathways and mediators [1,2,3]. It is frequently associated with metabolic disorders such as diabetes, dyslipidemia, non-alcoholic fatty liver disease, obesity, as well as hypertension, psoriatic arthritis and inflammatory bowel disease [4,5]. Adults with severe chronic plaque psoriasis are at elevated risk of mortality due to cardiovascular diseases [6,7]. This study focuses on five key biomarkers, namely, homocysteine, pentraxin 3, adiponectin, resistin, and leptin and their role in inflammation and metabolic regulation. These biomarkers were selected to investigate the inflammatory potential of psoriasis and its association with metabolic and cardiovascular risk in pediatric patients, as well as to assess their effectiveness in evaluating psoriasis severity. There are still only a small number of studies evaluating inflammatory markers in psoriasis in children, and there is a lack of standardized markers to assess the severity of the disease process in the pediatric population. Currently, the most commonly used instruments to assess inflammatory activity are based on visual assessment of the skin by using the psoriasis area and severity index (PASI) and body surface area (BSA). This subjective assessment of the severity of the disease may overlook the systemic inflammation in psoriasis patients; therefore, there is a need to find more objective biomarkers for assessing the severity of psoriatic lesions.

The PASI index has certain limitations and does not reflect the general state of inflammation in the patient’s body. Its result may be influenced by the use of local treatment and external conditions affecting the temporary condition of the skin during the examination; in addition, the PASI scale does not take into account the presence of psoriatic lesions in specific places, which, despite the small area of skin occupied, can significantly worsen the functioning of patients. Demonstrating an increase in markers indicating an inflammatory process in children may signify the risk of diseases accompanying psoriasis, which will allow for the implementation of necessary preventive measures. Homocysteine, a sulfur-containing amino acid and a metabolite of methionine, exacerbates inflammatory processes by activating Th1 and Th17 cells, promoting pro-inflammatory cytokine production and inducing oxidative stress. These mechanisms suggest its involvement in amplifying psoriatic inflammation [8,9,10,11]. Pentraxin 3, a glycoprotein classified as a long-chain pentraxin, is a key marker of systemic inflammation. Elevated pentraxin 3 levels have been reported in cardiovascular diseases, autoimmune disorders, and various inflammatory conditions [12]. Adipose tissue, being one of the body’s largest endocrine organs, secretes a wide range of biologically active substances known as adipokines; these include adiponectin, resistin, and leptin [13,14]. Adiponectin, primarily synthesized by adipocytes but also produced by cardiomyocytes and endothelial cells, has anti-inflammatory properties. It promotes the expression of anti-inflammatory markers, which may mitigate the inflammatory processes inherent to the disease [15]. Resistin, a peptide hormone predominantly secreted by monocytes and macrophages, both in adipose and non-adipose tissues, is highly responsive to pro-inflammatory cytokines such as IL-1β and IL-6. Its role in amplifying systemic inflammation makes it a potential marker for assessing severity of the inflammatory process [13]. Leptin, which is another adipokine primarily produced by adipose tissue, regulates energy homeostasis and immune response. Leptin is a biologically active protein that has pro-inflammatory effects by activating the secretion of inflammatory mediators. By reducing food intake and increasing energy expenditure through its action on hypothalamic receptors, leptin plays a crucial role in maintaining physiological energy balance. However, in obesity and chronic inflammation, hyperleptinemia and leptin resistance are frequently observed [16]. The purpose of this study was to assess the inflammatory potential of psoriasis and the risk of metabolic and cardiovascular diseases in patients with psoriasis by analyzing the serum concentrations of homocysteine, adiponectin, resistin, leptin, and pentraxin 3 in pediatric patients with psoriasis and in a control group. Moreover, determination of the relationship between the studied markers and the severity of psoriatic lesions was carried out.

## 2. Materials and Methods

### 2.1. Patients

This study included 103 patients. A total of 75 children with clinically diagnosed plaque psoriasis (47 girls and 28 boys), aged 2–17 years, were recruited from three pediatric dermatology centers between March 2020 and March 2022. These patients were treated exclusively with topical therapies. The control group consisted of 28 children (15 girls and 13 boys) selected from children without psoriasis and with non-inflammatory diseases in sequence reporting to the dermatology departments.

### 2.2. Clinical Assessment

Psoriasis severity in the study group was evaluated using the psoriasis area and severity index (PASI) and body surface area (BSA). Physical examinations, including body weight and height measurements, were performed to calculate the body mass index (BMI). On the basis of BMI and age, a patient was classified into normal (<85th and >5th percentile), overweight (≥85th and <95th percentile), or obese (≥95th percentile) categories.

### 2.3. Laboratory Analysis

Fasting venous blood samples were collected from participants, 8 mL in serum tubes and 4 mL in EDTA tubes. After centrifugation for 15 min at 1000× *g*, serum and plasma were aliquoted into Eppendorf tubes and stored at a temperature between −20 °C and −70 °C. Before the analysis, samples were thawed and homogenized. Serum concentrations of homocysteine, adiponectin, pentraxin 3, resistin, and leptin were measured using enzyme-linked immunosorbent assay (ELISA) kits according to the manufacturer’s instructions (QUANTIKINE, R&D Systems, Minneapolis, MN, USA, Bio-Techne). The concentration of homocysteine in the serum was expressed in µmol/L, while resistin was expressed in ng/mL, leptin was expressed in ng/mL, pentraxin 3 was expressed in pg/mL, and adiponectin was expressed in µg/mL.

### 2.4. Statistical Analysis

Statistical analyses were performed using Prism 9.0.1 (GraphPad Software Inc., La Jolla, CA, USA). Normality of the data distribution was assessed using the Shapiro–Wilk test. When data were non-normally distributed, logarithmic transformation (Y = log(Y)) was applied. If normality was not restored, nonparametric tests were used. For intergroup comparisons, the following methods were employed: (a) For normally distributed data, Student’s *t*-test for independent samples with Welch’s correction was applied in cases of unequal variances (assessed by the F-test). Data were presented as mean ± standard deviation. (b) For non-normally distributed data, the Mann–Whitney U test was used, whose results were expressed as medians and interquartile ranges. Effects of age (<10 years, ≥10 years) and sex (coded as 0/1) on biomarker levels were analyzed using three-way ANOVA, which included the main effects of age, sex, and group (psoriasis/control) as well as their interactions. Significant interactions were further explored using two-way ANOVA to isolate specific effects. Correlations between biomarker levels and clinical variables (PASI, BSA, age, sex, disease duration, pruritus, joint pain, obesity, family history of psoriasis) were assessed using Pearson’s correlation coefficient for normally distributed data or Spearman’s rank correlation coefficient for non-normally distributed data. Significant relationships (*p* < 0.05) were further quantified using effect sizes (e.g., Spearman’s rho). All statistical tests were two-tailed, and statistical significance was adopted for *p* < 0.05. Effect sizes were reported where applicable.

## 3. Results

### 3.1. Descriptive Characteristics of Participants

This study included 75 children with clinically diagnosed plaque psoriasis (47 girls, 28 boys; age range: 2–17 years, mean age: 8.5 years, standard deviation [SD]: 3.4) recruited from three pediatric dermatology centers. The control group consisted of 28 children (15 girls, 13 boys; mean age: 9.1 years, SD: 3.2) without psoriasis or other inflammatory/metabolic diseases, all with normal body weight.

**Figure 1:**
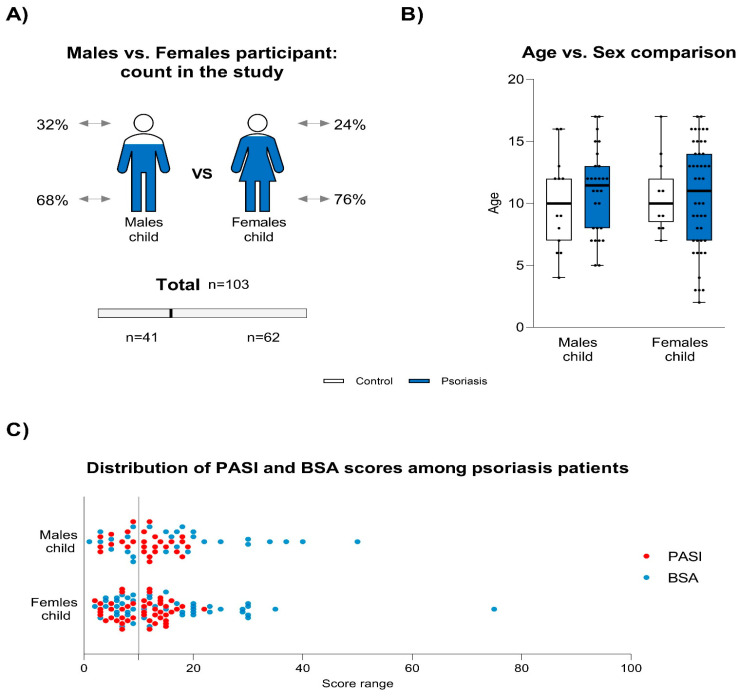
Demographic analysis of study participants and psoriasis severity scores. The figure presents a comprehensive overview of the demographics and clinical data of all 103 participants included in the study. (**A**) illustrates the distribution of male (n = 41) and female (n = 62) children between the control (outlined in white) and psoriasis (shaded in blue) groups. (**B**) shows a comparative analysis of age the distribution between male and female children across both the control and psoriasis groups, with similar age ranges observed in each category. The subfigure displays the interquartile range (IQR) along with minimum and maximum values, while the dots represent individual raw data points. (**C**) depicts the distribution of scores of the psoriasis area and severity index (PASI; red dots) and body surface area (BSA; blue dots) among the psoriasis patients, disaggregated by sex, highlighting the range of disease severity in male and female children. The grey line on the x-axis in (**C**) represents the threshold above which psoriasis severity was classified as moderate to severe.

In the psoriasis group, disease severity was assessed using the PASI score (range: 2–22, mean: 11, SD: 4.7) and BSA percentage (range: 1–75, mean: 17, SD: 12). Moderate to severe psoriasis (PASI > 10 or BSA > 10) was observed in 60% of the patients (Figure 1). Obesity (BMI ≥ 95th percentile) was identified in 13% (n = 10) of the psoriasis patients, while all children in the control group had normal body weight. A positive family history of psoriasis was noted in 41% (n = 31) of the psoriasis patients. Disease duration ranged from 1 month to 12 years, with a mean duration of 2.8 years (SD: 2.1).

### 3.2. Biomarker Levels

**Figure 2:**
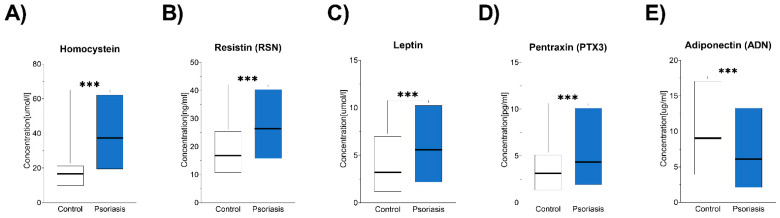
Comparative analysis of biomarker levels ((**A**). Homocystein, (**B**). Resistin, (**C**). Leptin, (**D**). Pentraxin, (**E**). Adiponectin) between psoriasis patients and controls. Floating bar plots display the minimum and maximum values, and bold lines represent either the mean (for homocysteine, resistin, leptin, and pentraxin 3) or the median (for adiponectin) in the psoriasis patients (blue) and controls (white). Statistical significance was assessed using Welch’s *t*-test for normally distributed data or the Mann–Whitney U test for non-normally distributed data, as appropriate. *** *p* < 0.001 indicates a highly significant difference between the groups.

Serum biomarker analysis revealed significantly higher levels of the following markers in the psoriasis group compared to the control group (Figure 2):

Homocysteine: Mean (±SD) 37.4 (9.1) µmol/L in the psoriasis group vs. 16.8 (3.8) µmol/L in the control group (*p* < 0.001, two-tailed; effect size = 0.73). This difference remained significant after making an adjustment for variance heterogeneity using the Welch-corrected Student’s *t*-test.

Resistin: Mean (±SD) 26 (6.8) ng/mL in the psoriasis group vs. 17 (3.8) ng/mL in the control group (*p* < 0.001, two-tailed; effect size = 0.31).

Leptin: Mean (±SD) 5.6 (1.7) ng/mL in the psoriasis group vs. 3.2 (1.4) ng/mL in the control group (*p* < 0.001, two-tailed; effect size = 0.31).

Pentraxin 3: Mean (±SD) 4.3 (1.4) pg/mL in the psoriasis group vs. 3.2 (0.9) pg/mL in the control group (*p* < 0.001, two-tailed; effect size = 0.17).

Conversely, significantly lower serum adiponectin levels were observed in the psoriasis patients compared to the control group; the median concentration was significantly lower in the psoriasis patients (6.1 µg/mL, range: 2.1–13 µg/mL) compared to the controls (9.1 µg/mL, range: 3.9–17 µg/mL; *p* < 0.001, effect size = 0.13).

The effect sizes suggest a large difference in homocysteine levels (effect size = 0.73), while differences in resistin, leptin, pentraxin and adiponectin represent moderate effects.

### 3.3. Correlation Between Resistin Levels and Psoriasis Severity

**Figure 3:**
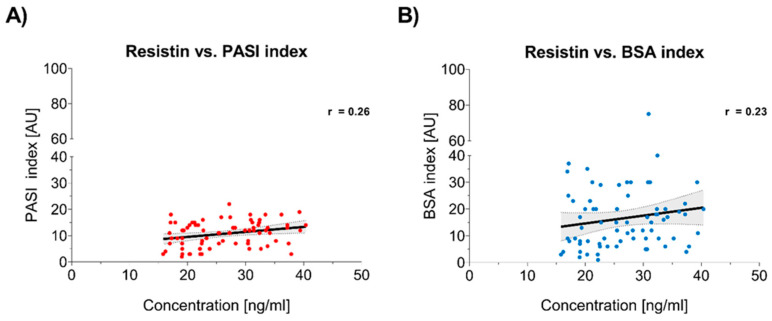
Resistin concentration in relation to clinical indices (PASI and BSA). The scatter dot plots demonstrate the association between resistin concentration (ng/ml) and two clinical indices: PASI and BSA (both in AU). In both panels, the black solid lines represent the regression lines, while the dotted lines indicate the 95% confidence intervals, providing an estimate of uncertainty. (**A**) Resistin vs. PASI index: A weak positive correlation was observed (Spearman’s r = 0.26, 95% CI: 0.030 to 0.47, *p* = 0.01). (**B**) Resistin vs. BSA index: A similar positive correlation was seen (Pearson’s r = 0.23, 95% CI: 0.0025 to 0.43, *p* = 0.02).

A weak but statistically significant positive correlation was found between resistin levels and psoriasis severity, as measured by the PASI (Spearman’s rho = 0.26, *p* = 0.01) and BSA (Spearman’s rho = 0.23, *p* = 0.02) (Figure 3). These results imply that resistin may reflect the extent of psoriatic inflammation.

### 3.4. Analysis of Other Factors

No significant associations were observed between biomarker levels and other clinical variables, including age, sex, positive family history of psoriasis, disease duration or obesity. Aggregated data for these analyses are summarized in Table 1 and Table 2.

## 4. Discussion

Psoriasis is recognized as a systemic inflammatory condition. Studies on adult population consistently point out its association with an increased risk of metabolic and cardiovascular diseases. Cytokines and pro-inflammatory substances, whose levels are elevated in psoriasis patients, play a significant role in the pathogenesis of these comorbidities. Studies on the association between psoriasis and other diseases are conducted on pediatric populations. It is highly important to understand this relationship and implement early preventive strategies because they could reduce the long-term burden of comorbidities in adulthood. Examining multiple biomarkers is significant for determining the contribution of each one in the disease pathogenesis and future usage in assessing disease activity, as well as in the selection of potential therapeutic options. In this study, we observed elevated levels of inflammatory markers such as homocysteine, pentraxin 3, resistin, and leptin as well as a reduced levels of anti-inflammatory adiponectin in pediatric psoriasis patients compared to healthy controls. In addition, we tested the usefulness of markers for assessing the severity of the disease. This could enable early intervention to modify the course of the disease and prevent comorbidities. The clinical usefulness of many biomarkers was studied, such as vitamin D. Its deficiency has been associated with the exacerbation of psoriasis when a trigger occurs, such as after COVID-19 vaccination [17].

Homocysteine is a mediator of inflammation, promoting Th1 and Th17 cell activation, increased production of pro-inflammatory cytokines, and oxidative stress. Hyperhomocysteinemia may increase the risk of atherosclerosis, cardiovascular diseases, venous thrombosis, stroke, and neurological conditions. Studies on adult psoriasis patients report elevated serum homocysteine levels. A Spanish study on 133 participants revealed higher homocysteine levels, metabolic syndrome, and carotid artery plaques in psoriasis patients compared to controls [18,19]. Similarly, a meta-analysis conducted by a Korean research team, comprising 16 studies and 2091 participants, confirmed significantly elevated homocysteine levels in psoriasis patients [19,20]. However, there are not enough studies in pediatric psoriasis patients. Our findings showed a significantly higher homocysteine levels in the children with psoriasis compared to the controls, suggesting that hyperhomocysteinemia may serve as an early marker of cardiovascular risk in this population.

Pentraxin 3 is a protein implicated in endothelial dysfunction and atherosclerosis. It is responsible for immune response, oxidative stress, and humoral innate immunity [21]. Produced by various cell types, including macrophages, fibroblasts, neutrophils, and endothelial cells, pentraxin 3 synthesis is primarily driven by inflammatory cytokines such as TNF-α and IL-1 [22,23]. Elevated pentraxin 3 levels have been observed in adult psoriasis patients. A Turkish study on 58 patients and an Italian study on 44 patients revealed significantly higher pentraxin 3 levels in psoriasis patients compared to controls. Our study similarly found significantly elevated pentraxin 3 levels in the children with psoriasis. These findings emphasize the inflammatory nature of psoriasis and its potential to increase the risk of other inflammatory and metabolic diseases in children [24,25,26].

Adipokines secreted by adipose tissue are known to promote inflammation and endothelial dysfunction as well as to impair glucose metabolism. Adiponectin is an anti-inflammatory adipokine that plays a crucial role in reducing inflammation. It is achieved by the promotion of macrophage regulation, the increase in the number of regulatory T cell populations (CD4+ Treg), the inhibition of pro-inflammatory cytokines such as TNF-α and IL-6, and the stimulation of IL-10 synthesis. In addition, adiponectin enhances insulin sensitivity and provides neuroprotective, anti-atherosclerotic, and cardioprotective effects [27,28,29]. Studies in adult psoriasis patients have revealed reduced adiponectin levels. An Indian study involving 60 patients and a Croatian study on 42 participants found significantly lower adiponectin levels in psoriasis patients compared to controls [30,31,32]. In our pediatric population, we observed a similarly reduced adiponectin levels, which points out the chronic inflammatory state in psoriasis and its potential role in reducing anti-inflammatory activity.

Resistin promotes the secretion of pro-inflammatory cytokines such as TNF-α and IL-12 and is involved in glucose metabolism, insulin resistance, endothelial dysfunction, and vascular smooth muscle proliferation. Resistin increases the expression of adhesion molecules and chemokine production in endothelial cells. These mechanisms contribute to its pro-atherogenic effects. Resistin is produced by monocytes/macrophages as well as some other cell types, including peripheral blood mononuclear cells and bone marrow cells. Resistin levels can be increased in obesity and other inflammatory diseases, but the correlation between the increase in BMI and resistin concentration is small. Resistin blood levels do not necessarily correlate with obesity and depend on the extent of inflammation [33,34,35]. In our study, we observed an increase in resistin levels in the psoriasis patients with both normal BMI and associated overweight and obesity.

Elevated resistin levels in adult psoriasis patients have been documented. A meta-analysis of 421 psoriasis patients conducted by Huang et al. showed significantly higher resistin levels [33,36]. An Iranian study aimed at determining a potential relationship between psoriasis with subclinical atherosclerosis, where psoriatic patients, in contrast to controls, demonstrated increased carotid intima-media thickness, which was accompanied by increased serum levels of resistin and leptin [37]. Our study similarly showed elevated resistin levels in the children with psoriasis, which confirms its role in predisposing pediatric patients to cardiovascular and metabolic complications.

In our study, the statistical analyses revealed an association between resistin levels and psoriasis severity, as measured by the PASI and BSA scores. Higher resistin levels were correlated with more severe disease, which implies that resistin is a potential marker of psoriasis severity. However, it is important to note that resistin alone is unlikely to be sufficient as a clinical indicator and should be interpreted in conjunction with other inflammatory and metabolic markers, as well as the overall clinical status of the patient. Bearing in mind that resistin plays a role in promoting pro-inflammatory pathways, its elevated level may also indicate an increased risk of other inflammatory diseases in patients with psoriasis. We would like to highlight that our correlation between resistin and PASI/BSA (R = 0.26, R = 0.23) was statistically significant but weak. Our observations were similar to those made by Coimbra et al. and Rajappa et al., who have shown a correlation between resistin and psoriasis severity. They found that resistin levels were higher in more severe cases of psoriasis [38,39]. A metanalysis by Kyriakou et al. showed a significant reduction in resistin levels in psoriasis patients after topical and systemic treatment, which may have clinical relevance in terms of using resistin to assess the severity of the inflammatory process [40]. Our study has certain limitations and drawbacks. The study population should be larger to draw conclusions. We did not evaluate in the study group whether the concentration of resistin decreased under the influence of treatment with decreasing the PASI index. The study was conducted in a pediatric population, where the condition and structure of the skin as well as the habits of the patients may differ in different age groups, which may interfere with a reliable comparison of the PASI and BSA indices in this group. Resistin appears to be a promising biomarker of psoriasis severity and may be useful for monitoring disease severity, risk of comorbidities in psoriasis, and effectiveness of treatment. We need reliable markers to assess disease severity and systemic inflammation in patients with psoriasis, which may help to better monitor and predict disease activity. Further studies are needed to determine the role of resistin and its correlation with clinical severity of psoriasis.

Leptin, another pro-inflammatory adipokine, induces the secretion of TNF-α, IL-1, and IL-6 and participates in macrophage activation, regulatory T cell modulation, and Th17 cell activity. These mechanisms contribute to the pathogenesis of inflammatory diseases, including psoriasis. In obesity, inflammatory cytokines stimulate leptin production, creating a vicious cycle of chronic inflammation [37,41,42,43]. Our findings demonstrated elevated leptin levels in pediatric psoriasis patients compared to controls, which corresponds to studies on adult populations [44]. A Taiwanese study found hyperleptinemia in psoriasis patients, regardless of obesity or metabolic syndrome [39,45]. However, other studies have noted elevated leptin levels in patients with psoriasis and coexisting obesity [46]. These complex interactions need further investigations.

In our study, the levels of the analyzed biomarkers in psoriasis patients were not related to body weight, which implies that psoriasis without accompanying obesity may be an independent risk factor for systemic inflammation. Resistin, in particular, appeared to be a potential marker of psoriasis severity, which could be used for monitoring disease progression. However, this relationship requires further investigation. The other studied substances did not correlate with disease severity, indicating that their elevated levels might represent an independent risk factor for the development of inflammatory and metabolic comorbidities in psoriasis patients, regardless of disease severity.

## 5. Conclusions

Psoriasis is a systemic inflammatory disease, which may increase the risk of other inflammatory, cardiovascular, and metabolic diseases, as is shown in the elevated levels of homocysteine, resistin, leptin, and pentraxin 3 as well as the reduced levels of the anti-inflammatory marker adiponectin in the psoriasis patients compared to the control group.

Effective treatment of skin lesions in children could not only improve the quality of life of patients but also reduce the risk of metabolic and cardiovascular comorbidities and may play a crucial role in reducing long-term mortality associated with these diseases in adulthood. Maintaining a proper weight, keeping a balanced diet, engaging in regular physical activity, and undergoing routine screening for comorbidities should become patient priorities.

The assessment of disease severity is influenced by many factors, so it is difficult to choose a marker that has real meaning in the clinic. Further studies are needed to investigate the relationship between selected metabolic and inflammatory biomarkers and psoriasis, with special consideration given to resistin as a marker of disease severity in larger cohorts of patients.

## Figures and Tables

**Table 1:** Analysis of relationships between biomarker levels, age, and sex in pediatric psoriasis patients and controls utilizing two-way ANOVA.

Biomarker	Effect of Group	Effect of Age	Effect of Sex	Interaction (Group × Age)	Interaction (Group × Sex)
Homocysteine	F = 45.67, *p* < 0.001 (Significant)	F = 1.12, *p* = 0.29 (ns)	F = 0.89, *p* = 0.35 (ns)	F = 0.72, *p* = 0.39 (ns)	F = 0.45, *p* = 0.50 (ns)
Pentraxin 3	F = 18.24, *p* < 0.001 (Significant)	F = 0.54, *p* = 0.46 (ns)	F = 0.62, *p* = 0.43 (ns)	F = 0.31, *p* = 0.58 (ns)	F = 0.23, *p* = 0.63 (ns)
Adiponectin	F = 22.18, *p* < 0.001 (Significant)	F = 0.92, *p* = 0.34 (ns)	F = 0.71, *p* = 0.40 (ns)	F = 0.56, *p* = 0.45 (ns)	F = 0.48, *p* = 0.49 (ns)
Resistin	F = 39.34, *p* < 0.001 (Significant)	F = 0.87, *p* = 0.35 (ns)	F = 0.53, *p* = 0.47 (ns)	F = 0.49, *p* = 0.49 (ns)	F = 0.36, *p* = 0.55 (ns)
Leptin	F = 27.89, *p* < 0.001 (Significant)	F = 0.67, *p* = 0.42 (ns)	F = 0.78, *p* = 0.38 (ns)	F = 0.41, *p* = 0.52 (ns)	F = 0.28, *p* = 0.60 (ns)

The table summarizes statistical results for the effects of group (psoriasis vs. controls), age, sex, and their interactions (group × age, group × sex) on biomarker levels. The results are reported as F-values and *p*-values for each factor and interaction terms across the five analyzed biomarkers: homocysteine, pentraxin 3, adiponectin, resistin, and leptin. Significant group effects (*p* < 0.001) were observed for all biomarkers, while no significant effects were found for age, sex, or their interactions (ns: not significant).

**Table 2:** Comprehensive comparisons of biomarker levels by demographic and clinical variables.

Variable	Biomarkers Tested	Statistical Test	Test Statistic (Range)	*p*-Value (Range)	Significance
Gender (Male/Female)	Homocysteine, PTX3, Adiponectin, Resistin, Leptin	Mann-Whitney U Test	U = 925.5–1056.0	0.23–0.68	ns
Family History	Homocysteine, PTX3, Adiponectin, Resistin, Leptin	Chi- square/Fisher Test	χ^2^ = 2.14–4.08	0.12–0.26	ns
Obesity (BMI ≥ 30)	Homocysteine, PTX3, Adiponectin, Resistin, Leptin	T-test/Mann- Whitney U	t = −1.13–0.94, U = 530.0–620.5	0.19–0.57	ns
Itching (Yes/No)	Homocysteine, PTX3, Adiponectin, Resistin, Leptin	Mann-Whitney U Test	U = 910.5–1025.5	0.21–0.63	ns
Joint Pain (Yes/No)	Homocysteine, PTX3, Adiponectin, Resistin, Leptin	Mann-Whitney U Test	U = 885.5–1012.0	0.18–0.59	ns

This table presents the results of statistical tests evaluating differences in biomarker levels (homocysteine, PTX3, adiponectin, resistin, leptin) across various demographic (gender, family history) and clinical variables (obesity, itching, joint pain) in pediatric psoriasis patients. For each test, the table presents ranges of test statistics and *p*-values obtained for the analyzed biomarkers. These ranges reflect the variability in the achieved concentrations for the different biomarkers within each tested group. The test results reveal that the differences were insignificant, which indicates that these variables do not have a substantial influence on biomarker concentrations.

## Data Availability

The data presented in this study are available on request from the corresponding author.
